# Delayed Effects of Corticosterone on Slow After-Hyperpolarization Potentials in Mouse Hippocampal versus Prefrontal Cortical Pyramidal Neurons

**DOI:** 10.1371/journal.pone.0099208

**Published:** 2014-06-05

**Authors:** Anup G. Pillai, Marloes J. A. G. Henckens, Guillén Fernández, Marian Joëls

**Affiliations:** 1 Dept. Translational Neuroscience, Brain Center Rudolf Magnus, University Medical Center Utrecht, Utrecht, The Netherlands; 2 Donders Institute for Brain, Cognition and Behaviour, Radboud University Nijmegen, Nijmegen, The Netherlands; 3 Dep. Cognitive Neuroscience, Radboud University Nijmegen Medical Center, Nijmegen, The Netherlands; University of Amsterdam, Netherlands

## Abstract

The rodent stress hormone corticosterone changes neuronal activity in a slow and persistent manner through transcriptional regulation. In the rat dorsal hippocampus, corticosterone enhances the amplitude of calcium-dependent potassium currents that cause a lingering slow after-hyperpolarization (sAHP) at the end of depolarizing events. In this study we compared the putative region-dependency of the delayed effects of corticosterone (approximately 5 hrs after treatment) on sAHP as well as other active and passive properties of layer 2/3 pyramidal neurons from three prefrontal areas, i.e. the lateral orbitofrontal, prelimbic and infralimbic cortex, with the hippocampus of adult mice. In agreement with previous studies, corticosterone increased sAHP amplitude in the dorsal hippocampus with depolarizing steps of increasing amplitude. However, in the lateral orbitofrontal, prelimbic and infralimbic cortices we did not observe any modifications of sAHP amplitude after corticosterone treatment. Properties of single action potentials or % ratio of the last spike interval with respect to the first spike interval, an indicator of accommodation in an action potential train, were not significantly affected by corticosterone in all brain regions examined. Lastly, corticosterone treatment did not induce any lasting changes in passive membrane properties of hippocampal or cortical neurons. Overall, the data indicate that corticosterone slowly and very persistently increases the sAHP amplitude in hippocampal pyramidal neurons, while this is not the case in the cortical regions examined. This implies that changes in excitability across brain regions reached by corticosterone may vary over a prolonged period of time after stress.

## Introduction

Exposure of an organism to stressful conditions causes the adrenal glands to release high amounts of corticosteroids (cortisol in primates, corticosterone in rats and mice). This hormone circulates in the body but also easily enters the brain where it binds to intracellular receptors to act as slow transcriptional regulators to modulate brain function [1], [2]. Two types of receptors have been identified: the mineralocorticoid receptor (MR), which due to its high affinity for corticosterone is already substantially activated under rest; and the lower-affinity glucocorticoid receptor (GR), which is particularly occupied after stress [2]. Furthermore, unlike the MRs, GRs are widely distributed in the brain, including in the hippocampus, amygdala nuclei, and prefrontal layers [3]–[5].

Despite the fact that GRs are rather ubiquitous in the brain, their role in modulating neuronal excitability has so far mostly been studied in the dorsal hippocampus. One of the most prominent effects of stress and corticosterone via CA1 hippocampal GRs is a slow and long-lasting enhancement in the amplitude of L-type calcium currents [6]–[9]. Downstream of the calcium influx, activation of calcium-dependent potassium channels may occur, in particular currents involved in the accommodation of firing frequency during periods of depolarization and the lingering slow after-hyperpolarization (sAHP) when the depolarization is terminated [10]. In line with this cascade, several studies have demonstrated that 1–4 hrs after administration of a brief pulse of corticosterone to pyramidal neurons in the rat dorsal CA1 hippocampal area, the amplitude of the sAHP is enhanced [11]–[13]; effects on firing frequency were somewhat more ambiguous [11], [13]. The modulation of sAHP amplitude by stress hormones might affect neuronal transmission and have important consequences for brain function such as learning and memory [14]–[16]. But most importantly, the alterations in sAHP amplitude by corticosterone in the aftermath of stress might be a crucial mechanism for normalizing the rapid increases of excitability observed soon after stress onset [17].

Interestingly though, the corticosteroid effect on the sAHP amplitude shows regional differentiation. In contrast to the enhanced sAHP amplitude observed in dorsal CA1 neurons several hours after corticosterone administration, principal cells in the basolateral amygdala (BLA) remain non-responsive [13] or even show the opposite effect [18]. Similar regional differentiation has been described with regard to morphological changes after chronic stress [19]. Neurons in the CA3 – and to a lesser extent CA1 – hippocampal area display reduced dendritic complexity after chronic stress [20]. This was also reported for neurons in the medial prefrontal cortex [21]–[23]. Yet, pyramidal neurons in the BLA [24] and orbitofrontal cortex (OFC) [25] display dendritic hypertrophy following chronic stress.

Given the *i)* regional differences in GR effects on sAHP amplitude in limbic regions and *ii)* the sensitivity of various regions in the frontal lobe to corticosterone and stress –albeit chronic-, we here studied the slow, presumably gene-mediated, effects of 100 nM corticosterone on AHPs, spike-frequency accommodation as well as other active and passive properties of pyramidal neurons in three prefrontal regions, i.e. the lateral OFC, the prelimbic cortex (PL) and the infralimbic cortex (IL). These were compared with GR-mediated actions in the dorsal CA1 neurons, as a positive control. In the OFC, PL and IL, we focused on pyramidal neurons in layers 2/3, which (like CA1 neurons) are a major source of efferent cortical projections and the primary center for intracortical processing [26]. To our knowledge, this is the first study that aims to compare the GR-mediated actions on both active and passive properties across various brain regions, using dorsal CA1 neurons as a positive control, under the same experimental conditions.

## Materials and Methods

For the present study we used 42 male C57/BL/6JOlaHsd mice (N = 12, 9, 18, and 10 mice for the dorsal CA1, lateral OFC, PL, and IL recordings respectively; in some mice more than one area was recorded) with an average age of 57 days. Mice were purchased from Harlan CPB, Zeist, The Netherlands, and socially housed (3–5 per cage) in a standard cage with enrichment (tissue paper & card board/plastic tubes) under a 12/12 h light/dark cycle (lights on at 7 am), with *ad libitum* access to food and water. All animals were acclimatized for at least one week before being used for the study. The experiments were approved and conducted with strict adherence to the guidelines of the Animal Committee for Bioethics (DEC) of the University of Utrecht (Permit number: 2011.I.08.081). Additionally, every effort was taken to reduce the number as well as the suffering of all experimental animals.

### Slice Preparation and Corticosterone Treatment

All mice were decapitated without anesthesia before 10.30 a.m. on the day of the experiment. This was necessary to keep the circulating glucocorticoid levels relatively low and uniform across experiments, as both the circadian cycle and anesthesia rapidly alter stress hormones levels in the plasma and brain [27]–[29]. The brain was quickly dissected out and placed in oxygenated (95% O_2_/5% CO_2_) ice-cold artificial cerebrospinal fluid (aCSF) of the following composition, in mM: 125 NaCl, 26 NaHCO_3_, 1.2 NaH_2_PO_4_, 10 Glucose, 3 KCl, 1.3 MgSO_4_ and 2 CaCl_2_ at pH ∼7.35. Subsequently, coronal sections of the brain, containing one or more of the regions of interest (hippocampus, orbitofrontal cortex, prelimbic, or infralimbic cortex), were cut at a thickness of 300–350 µm (PFC sections were cut at 300 µm to collect more slices) using a vibrating blade microtome (VT 1000S, Leica Biosystems, Germany). The slices were stored in a custom made slice holder containing oxygenated aCSF at room temperature (∼25°C). After a short period of recovery of ∼10–15 minutes, the slices were randomly split into two groups and transferred to one of two identical chambers (∼40 ml) filled with oxygenated aCSF at 30°C, containing either corticosterone (100 nM) or vehicle (0.01% ethanol) and incubated for 20 min. After the incubation, the slices were transferred back to the holding chamber containing normal aCSF at room temperature. The above method was adopted from a previous study where it was shown that this corticosteroid treatment is sufficient to observe the corticosteroid-induced persistent changes (after a delay of >1 hour) in cellular properties that require glucocorticoid receptor activation [7].

### Electrophysiology

For recording, one slice at a time was placed in the recording chamber of a patch-clamp setup while being continuously perfused with warm oxygenated aCSF (30°C, ∼2.5 mL/min; TC-324B, Warner Instrument Corp., USA) using a peristaltic pump. Neurons were visualized using a 40x objective (NA: 0.75, with Nomarsky optics IR-DIC) coupled to a b/w high resolution CCD camera and monitor (TCCCD-624 & CDM-1702, Monacor International, Bremen, Germany) attached to an AX10-Examiner (Zeiss, Germany) microscope. Whole-cell patch-clamp recording was carried out using an AxoPatch 200B amplifier (Axon Instruments, USA). The signals, sampled (at 50 kHz using Digidata 1322A, Axon Instruments, USA) and filtered (bandpass: 0–2 kHz), were acquired using the pClamp 9.2 software and analyzed off-line with custom programs written in Matlab.

Patch electrodes were made from thick-walled borosilicate glass capillaries (inner/outer diameter in mm: 1.5/0.86; Harward Apparatus, UK) pulled on a P-97 Flaming/Brown micropipette puller (Sutter Instruments, USA) to yield a tip of ∼2 µm (4–6 MΩ). The patch pipettes were filled with an intracellular solution that was composed of (in mM): 115 Potassium methanesulfonate, 20 KCl, 10 HEPES, 4 ATP-Mg^2+^ and 0.4 GTP-Na_2_ at pH ∼7.3 (adjusted with KOH). Calcium buffers were not included in the pipette solution as their presence is known to abolish both medium and sAHPs [30]–[32]. Unless stated otherwise, all chemicals were purchased from Sigma-Aldrich (The Netherlands).

In a limited number of cells we verified the location and shape of individual cells by including CF555 Hydrazide (50 µM; Sigma-Aldrich, USA) in the patch pipette. These slices were subsequently stained with DAPI (Life Technologies Corp., USA) and quickly imaged under a fluorescence microscope.

### Current Protocols

**Figure 1:**
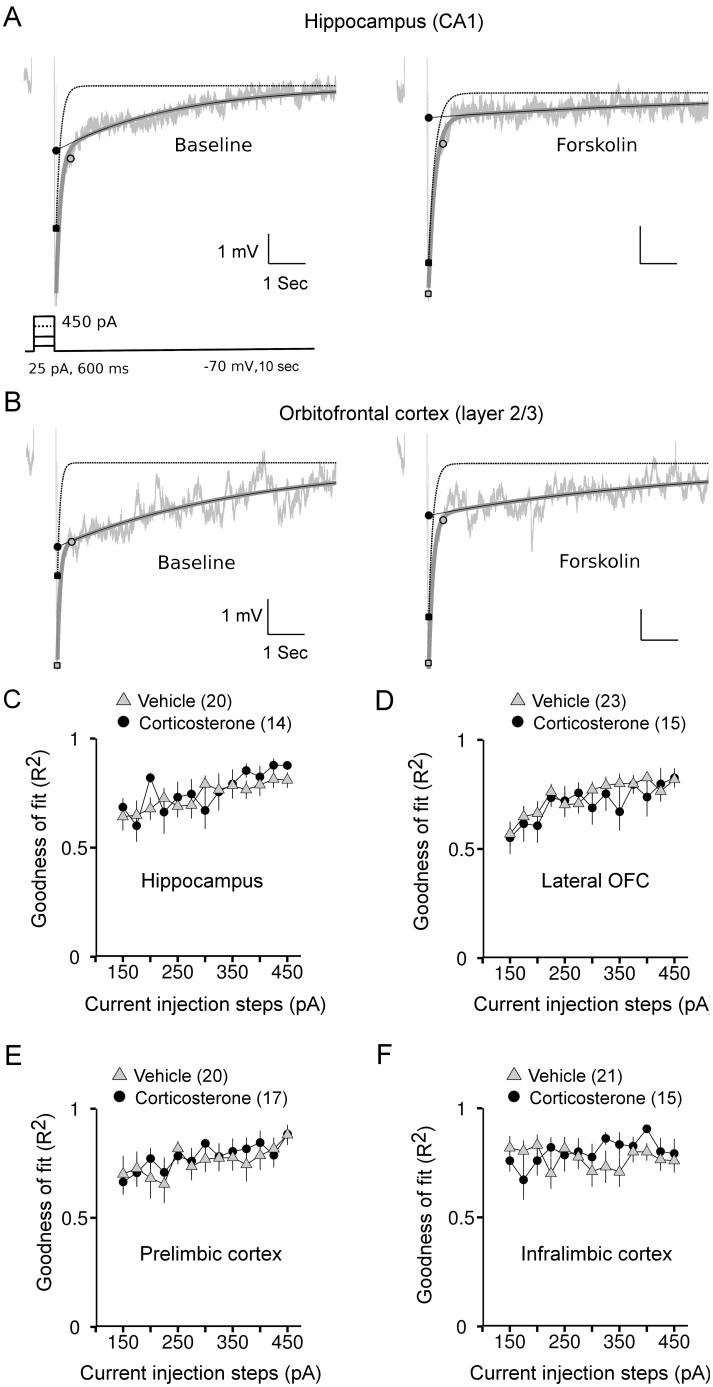
Bi-exponential fitting and pharmacological blockade of AHP responses in limbic pyramidal neurons. *A*: AHP recording from a representative CA1 neuron (gray) and the fit (dark gray) overlaid with the extracted decay of the medium (black-dotted) and sAHP (black-continuous) recorded from the same neuron before and after Forskolin (50 µm, 15 min) application. Inset shows the current protocol used to evoke the AHPs. *B*: AHP recording from a representative layer 2/3 lateral OFC neuron before and after Forskolin treatment. *C*: The goodness of fit (R^2^) to the AHP response is averaged across CA1 neurons recorded from the vehicle (gray diamond) and corticosterone (black circles) groups. *D*: Goodness of fit to the AHP response from layer 2/3 lateral OFC neurons. *E*: Goodness of fit to the AHP response from layer 2/3 prelimbic neurons. *F*: Goodness of fit to the AHP response from layer 2/3 infralimbic neurons. Number within brackets in the legends indicates number of neurons.

A holding current ensured that the cells were close to −70 mV before the start of the current protocol repeated at 30 s interval with increasing levels of depolarization in steps of +25 pA to reach a maximum of 450 pA. A short hyperpolarizing step (−20 pA relative to the holding current for 200 ms) was included to assess the passive membrane properties as well as series resistance. The depolarizing current step to initiate spike trains as well as the medium and sAHPs lasted for 600 ms and the responses were recorded for up to 8 seconds (see for more details Figure 1A). Additionally, an absolute zero current step (200 ms) was included at the end of each current sweep to check the membrane potential.

### Passive Membrane Properties

The resting membrane potential was inferred from the value of the membrane potential, at zero current, immediately after entering in whole-cell configuration. Passive membrane properties such as input resistance and membrane time constant were computed by fitting (Nelder-Mead Simplex) the filtered (linear squares regression, 1 ms window) trace with the following equation: V_p_ = V_offset_+(I_inj_*R_a_)*(1−exp(−t/tau_p_)+(I_inj_*R_in_)*(1−exp(−t/tau_m_), where V_offset_ is the membrane potential offset before current injection, I_inj_ is the injected hyperpolarizing current, R_a_, tau_a_, R_in_ and tau_in_ are the values of peak amplitude and decay time constant for the series and input resistance respectively. Tau_p_ refers to the lumped capacitance decay of the recording pipette. We additionally verified the reliability of the fitted values of input and series resistance by direct measurement from the raw trace for a subset of cells. Average series resistance of neurons recorded in different brain regions ranged between 25–33 MΩ and was not different between treatment groups.

We only targeted neurons with a pyramidal-like shaped cell body. However, selection of pyramidal-shaped neurons by visual means does not exclude the occasional recording from a cell belonging to a disparate group. This, however, is expected to be reflected in deviant membrane properties. We therefore included only cells that had an input resistance between 100 and 450 MΩ in the final analysis. This range was chosen to match the reported values from previous studies on pyramidal neurons in the hippocampus [33], lateral OFC [34] and infralimbic/prelimbic cortices [35].

### Action Potentials and AHP

For any given cell, measurement of active membrane properties was based on the first action potential and was averaged across current steps for each cell. Action potential threshold was calculated from the differentiated voltage trace where the rising slope of the spike surpassed 20 mV/ms. For every cell and for each current step, the peaks in the resulting voltage trace were counted as spikes only if the difference in amplitude was >40 mV from baseline membrane voltage. Action potential width at half maximum was measured as the interval at which the value of the membrane potential crossed half of the difference between the membrane potential at the peak and spike threshold during the rising and falling phase of the spike. Action potential rise time was calculated from the time difference at which the value of the membrane potential crossed 20% and 80% of the difference between the spike threshold and the peak amplitude. The slope of the action potential at the rising phase was computed by dividing the difference in membrane voltage at 80% and 20% of the spike height, as measured relative to the spike threshold, with the rise time. The ‘action potential peak amplitude’ mentioned in Table 1 indicates the absolute value of the membrane potential at the peak. Interspike intervals were computed in case of more than one spike and were averaged across spikes for each current injection step. Additionally, at each current step we also computed the ratio of the interval between the final two spikes and the interval between the first two spikes as a measure of spike-frequency accommodation [36].

**Table 1:** Active and passive properties of neurons within each of the limbic brain regions were not significantly different between treatment (vehicle and corticosterone) groups.

	Hippocampus (CA1)		Orbital frontal Cortex (layer 2/3)		Prelimbic Cortex (Layer 2/3)		Infralimbic cortex (layer 2/3)	
	Vehicle	Cort	Vehicle	Cort	Vehicle	Cort	Vehicle	Cort
**No. of cells**	20	13	21	14	20	17	21	15
**Resting membrane potential (mV)**	−71.6±0.9	−72.4±1.4	−75.1±1.5	−74.5±1.7	−73.0±1.6	−73.8±1.3	−74.1±1.0	−72.8±1.3
**Input resistance (MΩ)**	230.0±14	217.5±24	162.8±12	174.8±17	242.1±17	259.9±24	316.9±21	294.9±22
**Membrane time constant (ms)**	30.5±1.7	26±1.6	29.5±3.1	36.9±3.3	44.6±2.4	39.8±2.8	48.7±2.4	43.9±3.1
**Action potential amplitude (mV)**	60.0±1.3	57.0±1.5	54.7±1.4	54.8±1.4	54.9±1.4	54.8±1.2	54.7±0.7	55.0±0.8
**Action potential rise time (µs)**	167±10	152±5	156±7	166±9	180±7	185±6	200±9	199±10
**Action potential rising slope (mV/ms)**	423.9±19	423.8±15	355.1±14	341.5±15	313.3±18	305.6±15	290.0±14	298.0±19
**Action-potential threshold (mV)**	−50.4±1.0	−49.3±1.5	−36.9±1	−38.0±1.1	−35.8±1.3	−37.3±0.8	−36.9±0.7	−37.5±1.1
**Action potential width at half maximum (ms)**	1.49±0.05	1.42±0.04	0.94±0.05	0.99.±0.06	1.11±0.05	1.11±0.04	1.11±0.04	1.11±0.05
**mAHP decay (ms)**	105±10	122±17	254±17	214±22	228±22	239±20	174±20	197±23
**sAHP decay (sec)**	7.8±0.3	7.3±0.4	6.5±0.3	7.2±0.3	5.4±0.6	5.6±0.5	6.2±0.5	5.4±0.6

For the analysis of medium and slow after-hyperpolarization potentials, the raw data was first smoothed using linear least squares regression (window size: 1 ms). We separated the complex AHP signal into its two individual (the medium and slow) components using a nonlinear optimization technique where the decay of the total AHP amplitude was modeled as the sum of two individual exponentials (Figure 1) [37]. This was necessary to examine the distinct effects of corticosterone on medium and sAHPs. The fitting of the AHP decay also helped to make sure that noise fluctuations in the signal did not severely influence the measured AHP values, and to automatically determine kinetics of the sAHP. Thus, the following bi-exponential function was used to fit the decay of the AHP signal from the peak amplitude observed immediately after the end of the current step: V(t) = V_med_*exp(−t/tau_med_)+V_slow_*exp(−t/tau_slow_), where V_med_, tau_med_, V_slow_, tau_slow_, are the peak amplitude and decay of the medium and sAHPs respectively, while V(t) is the voltage at any time t. The fitting was performed using a Nelder-Mead Simplex algorithm that minimized the sum of squared errors between the fitted and actual trace with lower and upper bounds of 0–400 ms and 400 ms −8 s for the decay of the medium and sAHPs respectively. The constraints used in the fitting function were based on the values reported from other studies in the hippocampus for the decay of medium and sAHPs [38], [39].

We tested the validity of our methods regarding the estimation of the medium and sAHP parameters by applying a high concentration of Forskolin (50 µM, ∼15 min) in 3 cells from the hippocampus (Figure 1), where it is known to block the sAHP [40]. The peak amplitude of the mAHP fit, averaged for current steps between 325 and 450 pA, was largely unaffected by Forskolin (Baseline: −3.57±0.72 mV, Forskolin: −3.83±0.12 mV, 7% change, n = 3). However, values of peak amplitudes of sAHP, also obtained from the bi-exponential fit and averaged for the same current injection steps, were greatly reduced after Forskolin application (Baseline: −1.39±0.37 mV, Forskolin: −0.42±0.15 mV; 70% reduction). Additionally, the goodness of the bi-exponential fit for the AHPs as inferred from the Pearson correlation coefficient (R^2^) was on average close to 0.8 across all current injection steps in all brain regions examined, and approached 1 for higher current steps, where the AHP amplitude was also larger (Figure 1C–F).

### Statistical Analyses

All statistical analyses and plotting were carried out using the ‘R’ statistical software [41]. We used the *lme4* package [42] to perform linear mixed effects analysis on the effect of corticosterone on various parameters of AHP and action potential firing. The fixed effects in the model consisted of (1) a between group factor with two levels (treatment: vehicle and corticosterone), (2) a within group factor with 13 levels (current injections: 150 to 450 pA in steps of 25 pA) and (3) interaction between treatment and current injection. The model included random effects for intercept (to take into account the variability between neurons) and slope (to take into account the by-subject variability in the effect of current steps). Visual inspection of residual plots did not reveal any obvious deviations from homoscedasticity or normality. Significances were always obtained by likelihood ratio tests of the full model with the effect in question against the reduced model without the effect in question [43]. We took care of the missing data due to signal corruption by random noise at any current step from any cell using multiple imputation [44], [45]. This was necessary to be able to perform the two-way linear mixed-effects analysis with random effects. Multiple imputation was performed in R using the *Amelia II* package that employed the expectation maximization with bootstrapping algorithm to statistically impute the missing data [46], [47]. In our analysis, we combined over 500 imputations on the missing data to take into account any possible unexpected deviations on the imputed value [48]. We analyzed the data for any influential observation points [49] by computing the Cook’s distance from the linear mixed-effects model; this was performed in R using the *influence.ME* package [50]. Observation points that exceeded the cut-off value of 4/n, where ‘n’ is the number of observation points (value measured per current step from a given cell), were imputed as described above. Typical ‘n’ ranged from 350 to 450 (i.e. 13 current steps per cell x number of cells for each region) and the removed data points were between 5 and 15 per brain region (1–4%). Statistical power of the model was computed (for the lateral OFC only) from simulations (n∼1000) of data based on Markov chain Monte Carlo sampling from the posterior distribution of the parameter as obtained from the linear mixed-effects model.

## Results

### Corticosterone Treatment Persistently Increases sAHP Amplitude in Hippocampal CA1 Pyramidal Neurons without Changing Spike-frequency Accommodation

**Figure 2:**
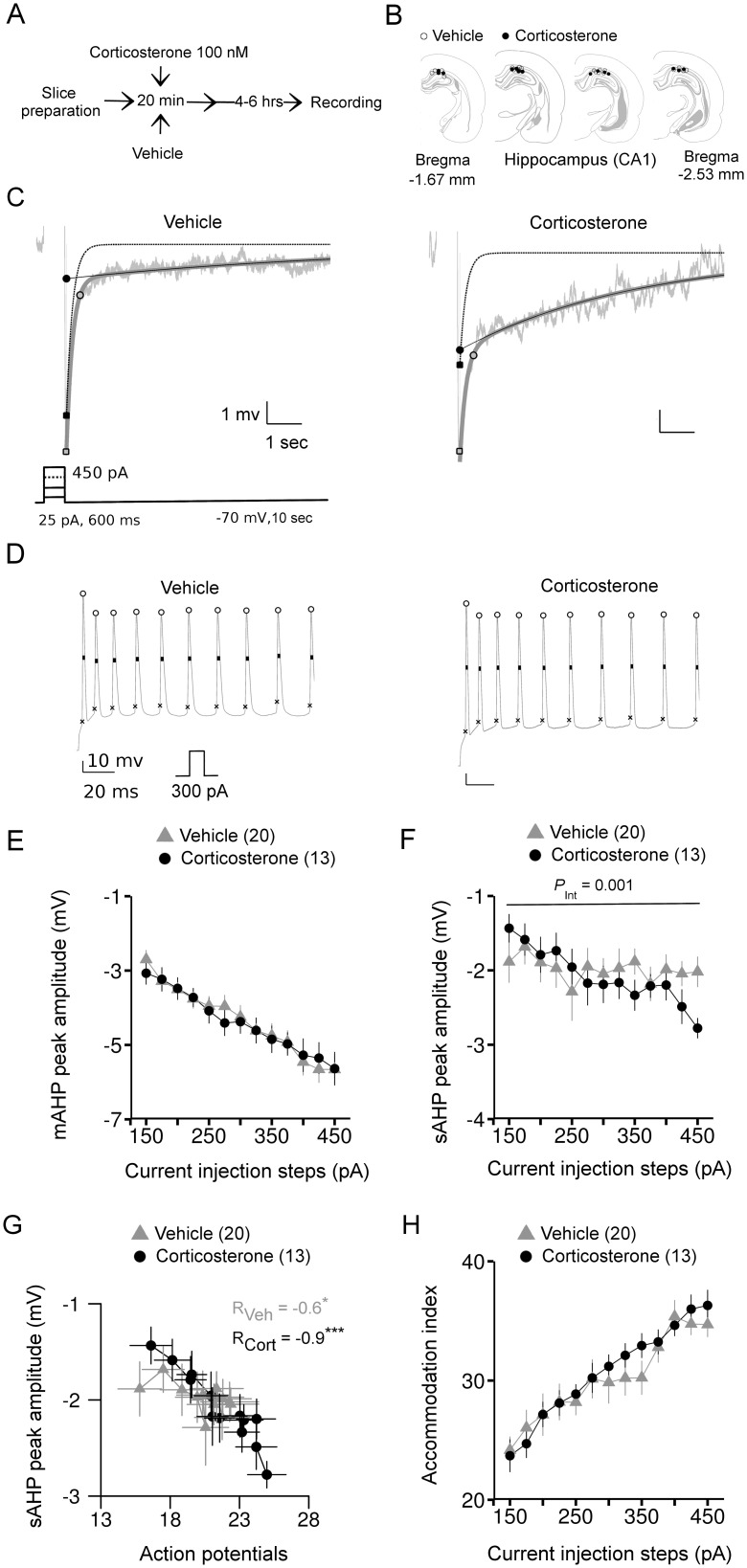
The long lasting effects of corticosterone treatment on AHPs and action potential firing of hippocampal CA1 pyramidal neurons. *A*: schematic depiction of the experimental protocol. *B*: locations of the recorded neurons on a pictorial coronal mouse brain section. *C*: representative AHP responses from vehicle and corticosterone groups overlaid with the fit. Light gray: raw trace, thick dark gray: fit, dotted line: mAHP decay, thin black line: sAHP decay. Inset shows the current protocol used to evoke the AHPs. *D*: traces of action potentials from neurons representative of the vehicle and corticosterone groups. Marking on the trace indicate the positions of spike amplitude, half-width and threshold measurements. *E*: mAHP peak amplitude averaged per group and per current step. *F*: averaged values of sAHP peak amplitude for each current injection step. *G*: sAHP peak amplitude is correlated with total number of action potentials per current step. R_Veh_/R_Cort_: Pearson’s correlation coefficient for vehicle and corticosterone treated groups. *: *P*<0.05; **: *P*<0.0001. *H*: accommodation index (% ratio of the interspike intervals of the last two spikes to the first two spikes) from vehicle and corticosterone groups. *P_Int_*: a significant interaction effect of corticosterone. Number within brackets in the legends indicates number of neurons.

Previously it was shown that the stress hormone corticosterone enhances the CA1 hippocampal sAHP amplitude after a delay of 1–4 hours, requiring glucocorticoid receptor activation as well as protein synthesis, thereby suggesting a genomic mechanism [6], [11], [13]. Therefore, we first sought to confirm these findings in the hippocampus. A delay of 4.5±0.3 hours was allowed between treatment and recording to increase the likelihood that the observed effects were the persistent, genomic-actions of corticosterone. Additionally, in all our recordings we targeted the pyramidal layer of the *dorsal* hippocampus, given the ventral-to-dorsal distinction in cellular effects of stress/corticosterone in the hippocampus [51]–[54]. A total of 33 pyramidal-shaped CA1 neurons that satisfied the selection criteria (100 MΩ<input resistance<450 MΩ) were analyzed to examine the delayed effects of corticosterone on both passive membrane and active intrinsic properties, including AHPs, action potentials, interspike intervals and spike-frequency accommodation (Figure 2A–H).

As summarized in Table 1, resting membrane potential, input resistance, and the membrane time constant were highly comparable for the vehicle and corticosterone-treated groups. Likewise, all properties of the action potential that were investigated were not noticeably affected by corticosterone.

In all of the recorded CA1 neurons, depolarizing current injections (starting from 150 to 450 pA, in steps of 25 pA and each lasting for 600 ms) evoked reliable AHPs that could be further distinguished into medium and slow components based on their decay kinetics. Accordingly, the bi-exponential function yielded good results on the fits for the AHPs evoked by each depolarizing current step (Figure 1C). The peak amplitude of the mAHPs, computed from successive current steps tightly overlapped between vehicle and corticosterone treated neurons, indicating no significant main effect of treatment (chi-square = 0.25, *P* = 0.6, ANOVA, Figure 2E) or its interaction with the injected current steps (chi-square = 1.29, *P* = 0.26, ANOVA). We next analyzed the peak amplitudes of sAHPs from the same neurons. As evident from figure 2C and F, there was a pronounced deviation towards increased sAHP amplitudes at higher current steps in the corticosterone treated cells. This was confirmed by a significant interaction effect of corticosterone treatment with the injected current steps (chi-square = 10.28, *P* = 0.001, ANOVA). Corticosterone had no major effect on the decay time (tau) of either medium or sAHP amplitude (Table 1).

Calcium entry during action potential firing is required for activating the calcium-dependent K^+^ channels that produce the after-hyperpolarization [55]. We therefore checked whether the increased sAHP amplitude in CA1 pyramidal neurons correlated to action potential number across the current injection steps. As expected, the sAHP amplitude in vehicle-treated neurons significantly correlated with the number of action potentials (Figure 2G, R = −0.6, *P*<0.05). Interestingly, the sAHP amplitude in corticosterone-treated neurons was even more strongly related to action potential number (R = −0.9, *P*<0.0001).

Conversely, AHP currents are important modulators of action potential firing in hippocampal pyramidal neurons, particularly the accommodation of firing frequency [55]–[57]. We therefore next focused on several active properties of action potentials in hippocampal neurons that are indicative of spike-frequency accommodation. The average number of action potentials for each current step did not differ between vehicle and corticosterone-treated cells (main effect: chi-square = 0.95 *P* = 0.32; interaction: chi-square: 1.39, *P* = 0.24, ANOVA, data not shown); nor did corticosterone alter the % ratio of the last spike interval with respect to the first spike interval, an indicator for spike-frequency accommodation, (Figure 2G, main effect: chi-square = 0.3 *P* = 0.6; interaction: chi-square: 0.96, *P* = 0.33, ANOVA). These results indicate a long lasting and delayed effect of corticosterone on sAHP amplitudes in CA1 neurons, but no noticeable effect in membrane properties or action potential firing.

### Corticosterone Treatment did not Alter the Amplitudes of AHPs in OFC Pyramidal Neurons

Exposure to stress influences OFC mediated behavior [58], [59]. Moreover, both chronic stress and corticosterone administration are known to induce structural changes in OFC neurons [60], [61]. However, the long lasting and delayed effects of acute corticosterone treatment on the intrinsic properties of layer 2/3 lateral OFC pyramidal neurons have not yet been tested. In this study, we explored both the passive membrane and active characteristics of 35 lateral OFC pyramidal neurons using whole-cell recording.

Similar to what we described previously for the hippocampus, the brain slices were incubated with either vehicle or corticosterone and left to recover for a period 5.4±0.4 hours before recording, in order to capture the delayed and persistent effects of corticosterone exposure. Passive membrane properties and characteristics of the action potential appeared not to be affected by corticosterone treatment (Table 1).

**Figure 3:**
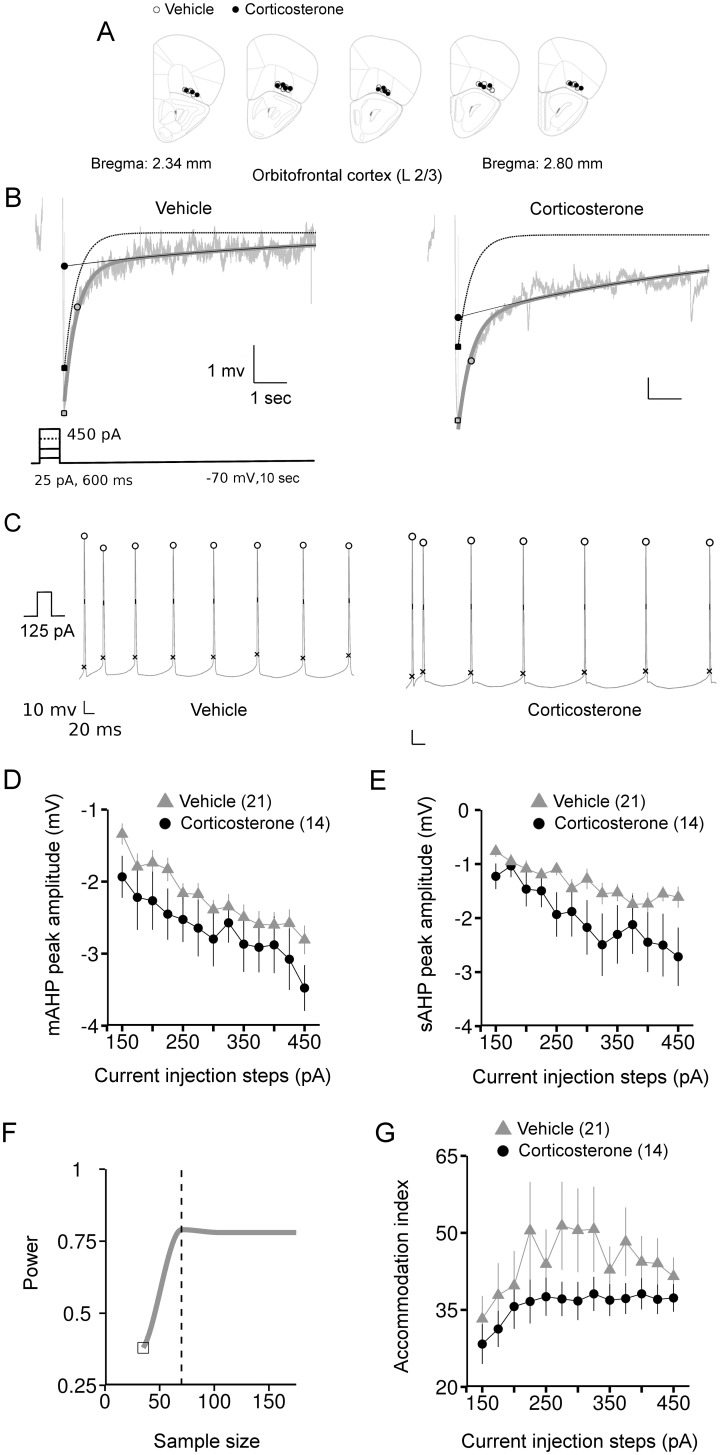
The long lasting effects of corticosterone treatment on AHPs and action potential firing of layer 2/3 lateral OFC pyramidal neurons. *A*: locations of the recorded neurons on a pictorial coronal mouse brain section. *B*: representative AHP responses from vehicle and corticosterone groups overlaid with the fit. Light gray: raw trace, thick dark gray: fit, dotted line: mAHP decay, thin black line: sAHP decay. Inset shows the current protocol used to evoke the AHPs. *C*: traces of action potentials from neurons representative of the vehicle and corticosterone groups. Marking on the trace indicate the positions of spike amplitude, half-width and threshold measurements. *D*: mAHP peak amplitude averaged per group and per current step. *E*: averaged values of sAHP peak amplitude for each current injection step. *F*: Power of the sAHP interaction effect (corticosterone treatment x current steps) with increasing total sample size. Dotted vertical line indicates the sample size (∼70) required for a power of 0.8. Open square in the plot indicates the power at the actual sample size. *G*: accommodation index (% ratio of the interspike intervals of the last two spikes to the first two spikes) from vehicle and corticosterone groups. Number within brackets in the legends indicates number of neurons.

Similar to the hippocampus, we observed an overlap in the amplitude of mAHP between cells treated with either corticosterone or vehicle (Figure 3D). There was neither a significant main effect of corticosterone (chi-square = 1.27, *P* = 0.26, ANOVA) nor an interaction with the injected current steps (chi-square = 0.054, *P* = 0.82, ANOVA, Figure 3D). Opposite to the hippocampus, corticosterone did not alter the amplitude of sAHP in the OFC (Figure 3E). Although the effect of corticosterone on sAHP amplitude became more pronounced at higher current injection steps, there was no significant interaction between treatment and current steps (chi-square = 2.96, *P* = 0.085, ANOVA, power = 0.41). To estimate the extent to which the sample size may have played a role in limiting the significance of this interaction effect in OFC neurons, we performed a *post hoc* power analysis (for details see Materials and Methods). This analysis revealed (Figure 3F) that an N of >70 neurons would be needed to obtain statistical power at the recommended 0.8 level [62]. We did not observe any effects of corticosterone on the kinetics of either mAHP or sAHP in OFC pyramidal neurons (Table 1).

We next examined the delayed and persistent effects of corticosterone on the active neuronal properties related to action potential firing and spike-frequency accommodation. Over the range of 150 until 450 pA, we did not observe changes in the number of action potentials per current step (*P*>0.2, data not shown), interspike intervals as well as the interval of the last spike in these neurons (*P*>0.4, data not shown). There was also no effect on the accommodation index as computed from the % of last to first spike interval ratio (Figure 3F).

### Corticosterone Treatment did not Alter the Passive Membrane or Intrinsic Cellular Properties in Layer-2/3 Pyramidal Neurons of the Prelimbic Cortex

**Figure 4:**
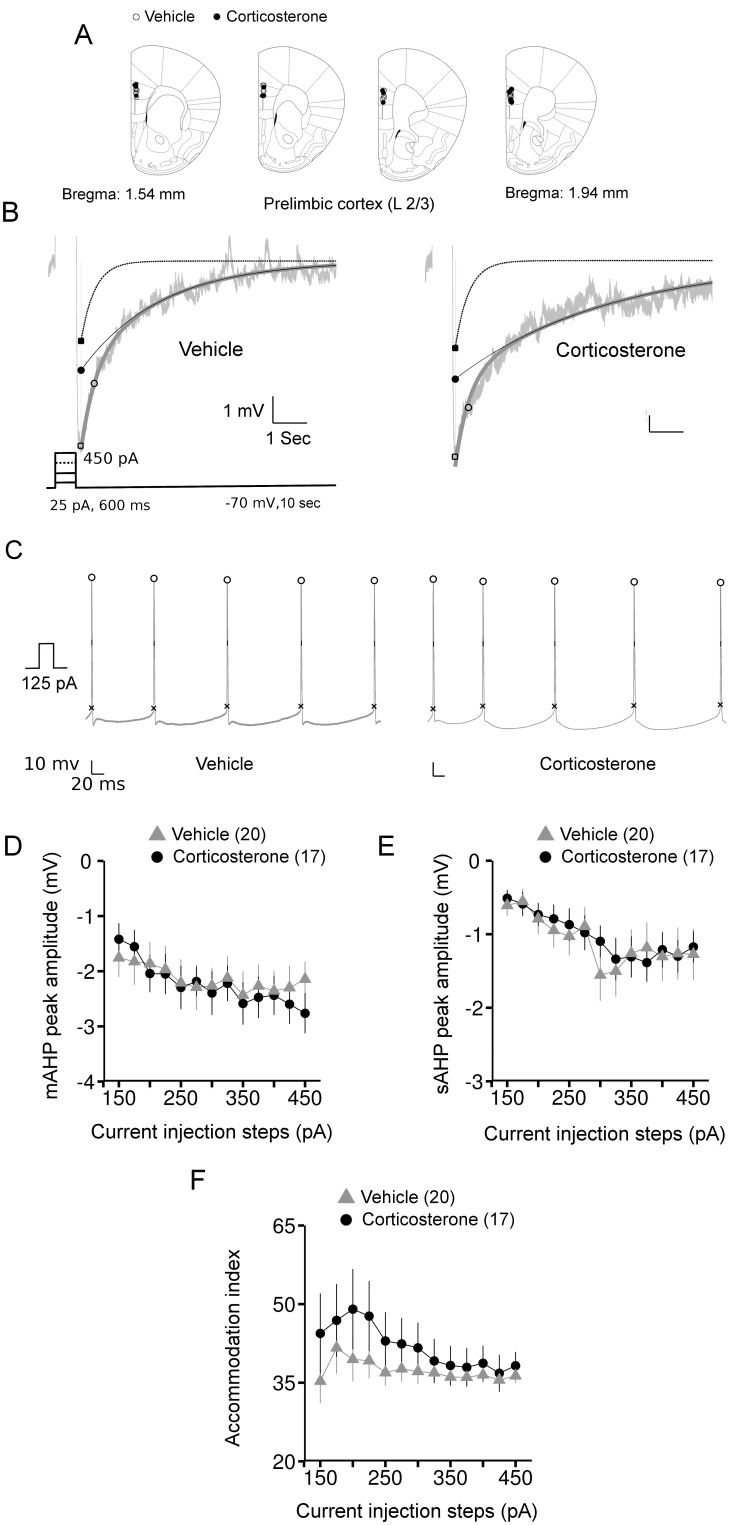
The long lasting effects of corticosterone treatment on AHPs and action potential firing of layer 2/3 prelimbic pyramidal neurons. *A*: locations of the recorded neurons on a pictorial coronal mouse brain section. *B*: representative AHP responses from vehicle and corticosterone groups overlaid with the fit. Light gray: raw trace, thick dark gray: fit, dotted line: mAHP decay, thin black line: sAHP decay. Inset shows the current protocol used to evoke the AHPs. *C*: traces of action potentials from neurons representative of the vehicle and corticosterone groups. Marking on the trace indicate the positions of spike amplitude, half-width and threshold measurements. *D*: mAHP peak amplitude averaged per group and per current step. *E*: averaged values of sAHP peak amplitude for each current injection step. *F*: accommodation index (% ratio of the interspike intervals of the last two spikes to the first two spikes) from vehicle and corticosterone groups. Number within brackets in the legends indicates number of neurons.

Both chronic stress and corticosterone treatment cause retraction of apical dendrites [62] and reduced number of spines [63] in layer 2/3 prelimbic (PL) neurons, emphasizing their sensitivity to prolonged exposure to glucocorticoids. We tested whether *acute* treatment with corticosterone can modify the membrane/intrinsic properties of PL neurons along with alterations in neuronal firing patterns. A total of 37 PL neurons were analyzed to examine the delayed and long-lasting effect of corticosterone treatment (Figure 4A–F). The average delay period between corticosterone treatment and recording was 4.8±0.5 hours. In line with the other brain regions we examined, characteristics of the membrane and action potential did not reveal differences between the corticosterone and vehicle-treated cells (Table 1). Depolarizing current pulses evoked considerable mAHP and sAHP amplitudes in the majority of PFC pyramidal neurons and these could be fitted with the bi-exponential function with fairly high goodness of fit values (Figure 1E and Figure 4B), although the amplitudes and signal-to-noise ratio were smaller in comparison to the CA1 neurons.

Peak mAHP amplitudes were not significantly affected by corticosterone treatment (Figure 4B and D, main effect: chi-square = 0.5645, *P* = 0.45) and similarly, there was no significant interaction with current steps (chi-square = 1.55, *P* = 0.21, ANOVA). In contrast to the hippocampus, but similar to OFC neurons, there was a complete absence of any delayed effect of corticosterone on sAHP amplitude in layer 2/3 pyramidal PL neurons (Figure 4E). This was evident from a lack of any significance for either the main effect (chi-square = 0.53, *P* = 0.47) or interaction (chi-square = 0.06, *P* = 0.8, ANOVA) of corticosterone treatment with current steps.

Furthermore, no effect of corticosterone could be discerned on any parameters of action potential firing or spike-frequency accommodation in layer 2/3 PL neurons (Table 1 and Figure 4C). Nor did we observed any significant effects of corticosterone on spike-frequency accommodation (main effect: chi-square = 1.28, *P* = 0.26; interaction: chi-square = 1.32, *P* = 0.25, ANOVA, Figure 4F).

### AHP Amplitudes of Layer-2/3 Pyramidal Neurons in the Infralimbic Cortex were not Affected by Corticosterone Treatment

The IL area of the PFC has recently gained considerable attention from studies that have associated its activation to reduced stress sensitivity or resilience to stressful insults [64], [65]. Therefore, it was of interest to us to examine the role of glucocorticoids in the modulation of both the mAHP and sAHP in this area, and specifically in the pyramidal neurons of layer 2/3, as they undergo major structural alterations after prolonged exposure to either stress [21], [65] or corticosterone [66].

A total of 36 pyramidal neurons from layer 2/3 of the IL, recorded several hours (4.5±0.2 hours) after either vehicle or corticosterone treatment, were included in this study. Similar to other brain regions we examined, there was no persistent effect of corticosterone treatment on either the passive membrane or action potential properties (Table 1).

**Figure 5:**
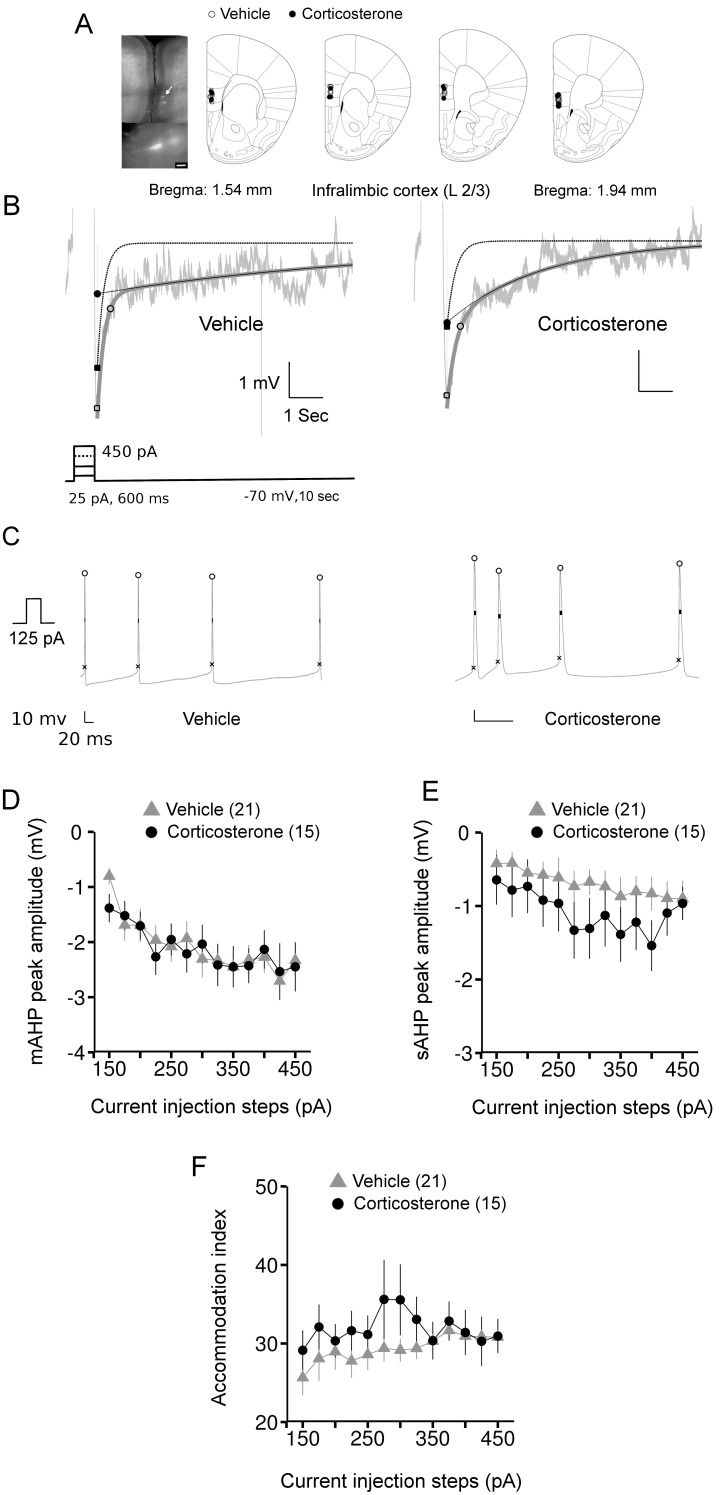
The long lasting effects of corticosterone treatment on AHPs and action potential firing of layer 2/3 infralimbic pyramidal neurons. *A*: *left*: a sample pyramidal neuron filled with fluorescent dye. Inset: zoomed image of the neuron (white arrow). Scale bar equals 20 microns. *Right*, locations of the recorded neurons on a pictorial coronal mouse brain section. *B*: representative AHP responses from vehicle and corticosterone groups overlaid with the fit. Light gray: raw trace, thick dark gray: fit, dotted line: mAHP decay, thin black line: sAHP decay. Inset shows the current protocol used to evoke the AHPs. *C*: traces of action potentials from neurons representative of the vehicle and corticosterone groups. Marking indicate the positions of spike amplitude, half-width and threshold measurements. *D*: mAHP peak amplitude averaged per group and per current step. *E*: averaged values of sAHP peak amplitude for each current injection step. *F*: accommodation index (% ratio of the interspike intervals of the last two spikes to the first two spikes) from vehicle and corticosterone groups. Number within brackets in the legends indicates number of neurons.

The characteristics of both the mAHP and sAHP were estimated from the bi-exponential fit (R^2^>0.8, Figure 1F). A striking overlap in the values of mAHP was observed between the vehicle and corticosterone groups at all the current steps (Figure 5D). Accordingly, no significant effects were evident for both main and interaction effect of corticosterone (main effect: chi-square = 0.54, *P* = 0.46; interaction: chi-square = 0.42, *P* = 0.52, ANOVA). Changes in the averaged values of sAHP peak amplitudes also did not reach statistical significance (main effect: chi-square = 0.54, *P* = 0.46; interaction: chi-square = 0.02, *P* = 0.89, ANOVA, Figure 5E).

No major effect of corticosterone treatment was obvious in various parameters of action potential firing such as number of action potentials, average interspike interval and the last spike interval (*P*>0.5, ANOVA, data not shown). Moreover, the % ratio of the last spike to first spike interval, an index for spike-frequency accommodation, was also similar between corticosterone and vehicle treated neurons (Figure 5F, main effect: chi-square = 1.81, *P* = 0.2; interaction: chi-square = 1.19, *P* = 0.27, ANOVA).

## Discussion

The slow gene-mediated effects of corticosterone or stress on the brain, presumably involving the activation of GRs, are well-documented (reviewed in [17]). Despite the moderately high expression levels of GR in cortical layers [67], including the OFC and medial prefrontal cortex [68], corticosteroid actions in these regions have been understudied, particularly in layer 2/3 which is the main intracortical processing layer of the prefrontal cortex [69], [70]. We here report that exposure to corticosterone, at a concentration that is sufficiently high to activate GRs [7], failed to affect the amplitude of the sAHP in pyramidal cells of the lateral OFC as well as the PL and IL regions of PFC. Our results indicate that the slow and presumably gene-mediated effects of corticosterone on sAHP as earlier shown for sub-cortical areas such as the CA1 and CA3 areas of the hippocampal formation [11]–[13] and the basolateral amygdala [18] are highly region dependent. Other properties of hippocampal, lateral OFC and medial prefrontal neurons, more specifically in layer 2/3 pyramidal neurons, be it active or passive, were not affected by the stress hormone.

### Effects in Hippocampus

In agreement with earlier findings in the hippocampus [9], [11]–[13], we here did find a significant effect of corticosterone on the sAHP amplitude, which became more prominent with increasing current injection steps. Despite this enhancing effect of corticosterone on the sAHP amplitude, we did not find notable effects on parameters indicative of spike-frequency accommodation in CA1 pyramidal neurons; this suggests that other mechanisms are possibly in play. Corticosterone was earlier reported to have a delayed effect on hippocampal firing [11], although the effects were generally not strong [13]. The current study differed from earlier ones in the extent of the delay between corticosterone exposure and recording. In previous studies, this delay ranged from 1 to 4 hrs [11], [13], but in our study the delay on average was well over 4 hrs. We introduced this extended time-window to fully exclude the influence of any fast non-genomic actions and focus exclusively on slowly-developing and very persistent genomic actions. We cannot exclude that after such an extended delay, the effects of corticosterone on sAHP might start to subside and possibly, the effects of corticosterone on other channels determining firing frequency (e.g. I_A_) might start to compensate for the changes in calcium dependent K^+^-conductances that contribute to the sAHP [71]–[74]. Indeed, a few studies have already reported that a complete or partial blockade of sAHP does not invariably and appreciably influence interspike intervals suggesting that the currents underlying spike-frequency accommodation in CA1 pyramidal neurons may not be limited to those responsible for the sAHP [55], [75]. Thus, while there is consensus that a major source of external calcium required for AHPs is from the L-type calcium channels [77] –which are affected by corticosterone [78], [79]- calcium from other sources, such as through activation of NMDARs or the hyperpolarization-activated cyclic nucleotide gated h-current [80] following repetitive firing, as well as their co-localizations with the sAHP channels might also contribute to the effects we observed. We also observed that the correlation between the number of action potentials and the sAHP amplitude was enhanced after corticosterone compared to vehicle treatment. Therefore, although corticosterone did not increase the total number of spikes per current injection step, the same spikes after treatment were able to elicit larger sAHP responses, particularly with the higher current injection steps.

Similar to the effects of corticosterone on spike trains, properties of single action potentials were also comparable for vehicle- and corticosterone-treated neurons. Notably, the limitations of the typical patch-clamp amplifier, when in the current-clamp mode as used in this study can contribute to significant errors in the measurement of active properties [76]; this is very often completely ignored. Despite this limitation, we have no clear indications (now or in earlier studies) that corticosterone slowly changes active membrane properties in CA1 neurons.

### Effects in Prefrontal Areas

To our knowledge this is the first study that compares slow corticosteroid-dependent actions in electrical properties across a variety of areas, using the exact same experimental conditions (and in some cases even slices from the same animal). In contrast with the hippocampus, we did not find an increase in sAHP amplitude after corticosterone treatment in the lateral OFC, PL or IL layer 2/3 pyramidal neurons [77]. Although the sample size for all prefrontal areas was comparable to that of the hippocampus, the effect size was much smaller; in order to reach significance we would have needed to considerably increase the number of cells. Along with this lack of change in the sAHP amplitude, we also did not observe any significant effects on spike firing accommodation in OFC, PL and IL neurons. Our results might be seen as surprising, given that both the prelimbic and infralimbic regions have been reported to be very sensitive to stress or corticosterone exposure. Even shifts in the amount of corticosterone released during the diurnal rhythm by itself are sufficient to induce alterations in dendritic length [22] and spine density [77] in layer 2/3 IL neurons [77]. Stress sensitivity can also be inferred from an *in vivo* study, in which stress-induced alterations in both PL and IL neuronal activity were found to be correlated with a deficit in extinction retrieval after stress episodes [78]. However, as earlier mentioned, there is a severe lack of detailed studies on AHP in PFC neurons and lack of knowledge of the channel subtypes as well as downstream mechanisms that are involved in mediating both medium and slow AHPs in these neurons. In fact, not only are the underlying action potential properties different between the hippocampus and PFC neurons, so are the kinetics of the medium and slow AHPs (Table 1). We further cannot rule out the possibility that ion channels responsible for the sAHP might exhibit an age-dependent decline as was found to be the case with BK-type calcium-dependent potassium channels in the PFC regions [79].

In the light of our current understanding, it is difficult to speculate on the differences in mechanisms that could have led to the absence of any noticeable corticosterone effect on AHPs and action potential firing in IL, PL and OFC neurons. It should be noted that neurons in these regions are not completely unresponsive to corticosterone. Thus, corticosterone was found to affect glutamatergic transmission in PFC neurons and the effects were very similar for PFC and hippocampal cells [80], [81]. These studies have suggested a role for serum- and glucocorticoid-inducible kinase and Rab4 activation to result in the facilitation of Rab4-mediated recycling and insertion of AMPA receptors into the plasma membrane. The presence of a highly comparable slow, GR-mediated increase in both AMPA receptor surface expression and amplitude of spontaneous AMPA-receptor mediated postsynaptic currents in hippocampal neurons of adult mice suggests that some commonality might exist in the downstream targets of corticosterone, or in at least some of its lasting effects on excitability [82]–[84].

However, there are also studies that point towards regional differences between the hippocampus and cortical neurons. For instance, nuclear translocation of GR was found to be of higher amplitude in prefrontal cortical tissue than in the hippocampus [85]. Additional differences may even arise further downstream, i.e. after transcriptional regulation, due to a process between transcriptional and translational control of GR-responsive genes [86] or depend on local expression of ion channel subunits or intracellular proteins [13]. Possibly the involvement of a different receptor for corticosterone could explain the differences in sAHP between brain regions. While PL neurons do express moderately high levels of GR whose activation is necessary for the increase in sAHP amplitude in CA1 neurons [11], [12], they have far less MR expression and this contrasts with the hippocampal CA1 sub-field where it is in considerable abundance [67].

Unlike stress and corticosterone effects on glutamatergic transmission in layer 5 PL neurons [80], [81], [87], the effect of corticosteroid hormones on electrical activity of neurons in the OFC is mostly unknown [88]. We here focused – in addition to the PL and IL- also on neurons in the lateral OFC, based on their sensitivity to (chronic) stress in terms of structural changes [21]–[23], [60]. OFC pyramidal neurons – similar to BLA principal cells [24] – show expansion of their dendritic tree after chronic stress [25], which contrasts with reports on neurons in the medial prefrontal cortex and hippocampal CA3 and (to a lesser extent) CA1 neurons [20]–[23].

In summary, the present study (together with previous work) indicates that a wave of corticosterone, as occurs in response to stress, changes particularly the sAHP amplitude in a region-dependent manner, leaving other passive and active membrane properties (as far as tested) generally unaffected. These delayed effects, presumably gene-mediated, may subsequently lead to region-dependent changes in information transfer following stress through the dorsal hippocampus, ventral hippocampus [51], [52], [89], BLA [13], [18], OFC, PL, and IL. Thereby, the overall effect of stress on the functioning of brain circuits is a complex composite of various distinct local actions.
